# The History of *Mycoplasma pneumoniae* Pneumonia

**DOI:** 10.3389/fmicb.2016.00364

**Published:** 2016-03-22

**Authors:** Takeshi Saraya

**Affiliations:** Department of Respiratory Medicine, Kyorin University School of MedicineMitaka, Japan

**Keywords:** *Mycoplasma pneumoniae* pneumonia, Eaton agent, Pinehurst trials, primary atypical pneumonia, history

## Abstract

In the United States in the 1930s, although the pathogen was not known, atypical pneumonia was clinically distinguished from pneumococcal pneumonia by its resistance to sulfonamides. Reimann (1938) reported seven patients with an unusual form of tracheo bronchopneumonia and severe constitutional symptoms. He believed the clinical picture of this disease differed from that of the disease caused by influenza viruses or known bacteria and instead suspected “primary atypical pneumonia.” For many years, the responsible infectious agent was tentatively classified as a filterable virus that could pass through a Seitz filter to remove bacteria and was reported to be a psittacosis-like or new virus. After that, Eaton et al. (1942, 1944, 1945) identified an agent that was the principal cause of primary atypical pneumonia using cotton rats, hamsters, and chick embryos. Eaton et al. (1942, 1944, 1945) did not perform an inoculation study in human volunteers. During the 1940s, there were three groups engaged in discovering the etiology of the primary atypical pneumonia. (1) Commission on Acute Respiratory Diseases Diseases directed by John Dingle, (2) Dr. Monroe Eaton’s group, the Virus Research Laboratory of the California State Public Health Department, (3) The Hospital of the Rockefeller Institute for Medical Research directed by Horsfall. During 1940s, the members of the Commission on Acute Respiratory Diseases concluded that the bacteria-free filtrates obtained from the patients, presumably containing a virus, could induce primary atypical pneumonia in human volunteers via Pinehurst trials. During 1950s, serological approaches for identification of the Eaton agent developed such as Fluorescent-Stainable Antibody, and at the beginning of the1960s, the Eaton agent successfully grew in media, and finally accepted as a cause of primary atypical pneumonia. Thus, technical difficulties with visualizing the agent and failure to recognize the full significance of the Pinehurst transmission experiments resulted in a lapse of 20 years before acceptance of the Eaton agent as *Mycoplasma pneumoniae*. This review describes the history of *M. pneumoniae* pneumonia with a special focus on the recognition between the 1930 and 1960s of the Eaton agent as the infectious cause.

## Introduction

Atypical bacterial pneumonia is caused by atypical organisms that are not detectable on Gram stain and cannot be cultured using standard methods, and characterized by a symptom includes headache, low-grade fever, cough, and malaise. The most common organisms are *Mycoplasma pneumoniae*, *Chlamydophila pneumoniae*, and *Legionella pneumophila.* The history of *C. pneumoniae* began in Taiwan in 1965, which was first isolated from the eye of a child in a trachoma vaccine study and first isolated from the respiratory tract in 1983 from a University of Washington student (Grayston et al., 1986; Grayston, 2000). Among them, *M. pneumoniae* is one of the leading causes of community acquired pneumonia. The term mycoplasma emerged in the 1950s and means “mykes” (fungus) and “plasma” (formed) in Greek. Isolation of the first mycoplasma was the bovine pleuropneumonia agent, now known as *M. mycoides* subsp. *mycoides*, which was reported initially in Nocard and Roux (1898). This bacterium became to know over the next 50 years as pleuropneumonia-like organisms (PPLO) in various animals. Dienes and Edsall (1937) detected first *Mycoplasma* isolated from humans in a Bartholin’s gland abscess, known as *M. hominis*. Regarding with *M. pneumoniae*, it was first isolated in tissue culture from the sputum of a patient with primary atypical pneumonia by Eaton et al. (1944) as Eaton agent. However, its taxonomy remained obscure until the early 1960s when it was clearly identified as a bacterium. The cell volume of *M. pneumoniae*, is less than 5% of that of a typical bacillus and rarely exceed 100 μm in diameter. *M. pneumoniae*, lacks a cell wall, which makes it intrinsically resistant to antimicrobials, such as β-lactams. In this regards, identification of the *M. pneumoniae* was a challenging issue for pioneers. This review focus on the history of discovering and acceptance the Eaton agent as the cause of primary atypical pneumonia.

## Atypical Pneumonia-Discovery of a New Clinical Syndrome (1940s)

Reimann (1938, 1984) reported several patients with similar clinical features such as mild symptoms of hoarseness, sore throat, pyrexia with relative bradycardia, and persistent dry cough. The fever lasted from 10 to 43 days in the cases of severe involvement but most typically only lasted about 3 weeks. He believed that those symptoms were strikingly similar to those of patients in a report by Scadding (1937) from London, characterized as gradual onset, malaise, shivering, dyspnea, dry cough, marked sweating, slight leukocytosis, and roentgenographic shadows of diffuse pneumonia. Reimann also indicated that colleagues in other East Coast cities had recognized this syndrome, but it was usually diagnosed as influenza.

Indeed, Meiklejohn et al. described primary atypical pneumonia as being caused by psittacosis-like viruses (Meiklejohn et al., 1944) and/or a new atypical pneumonia virus (Meiklejohn et al., 1945). Around the same time, Dingle described that primary atypical pneumonia of unknown etiology was a more common disease than previously thought (Finland and Dingle, 1942).

## Discovery of the Eaton Agent and Associated Animal Models

**FIGURE 1:**
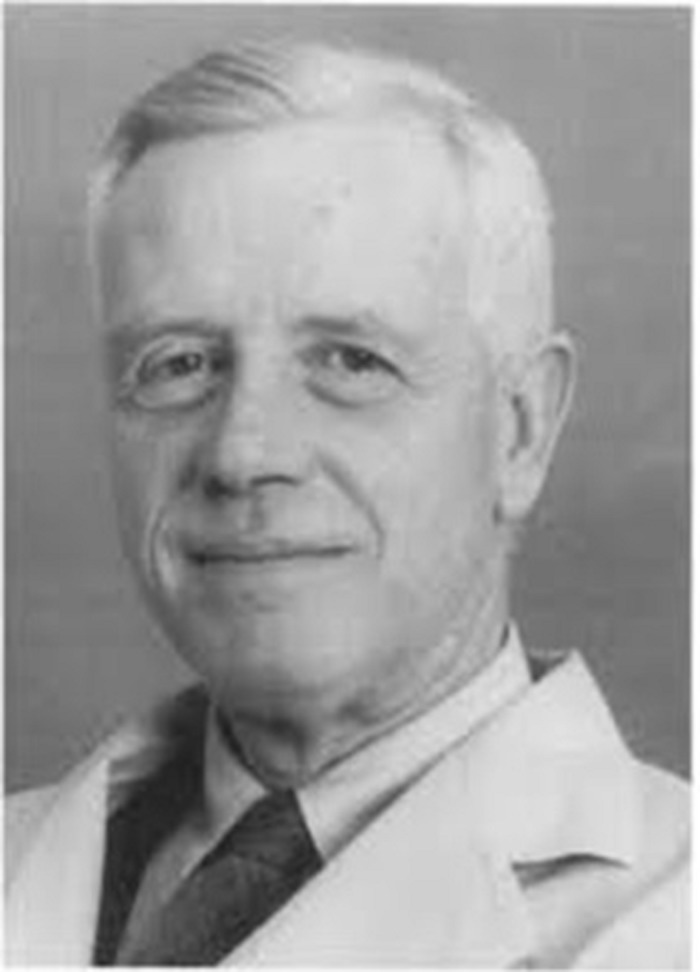
**Photograph of Dr. Eaton Eaton, Monroe D., U.S. microbiologist, 1904–1958.** The photograph of Dr. Eaton in the manuscript (*Rev Infect Dis*, 12, 338–353), which taken in the early 1960s and reprinted permission was obtained.

Eaton et al. (1942) (**Figure 1**) also reported that an infectious agent obtained from a total of 78 patients with atypical pneumonia was apparently transmissible to cotton rats. Most of the inoculation materials were retrieved from sputum or lung samples from patients with atypical pneumonia and were intranasally inoculated to the cotton rats. Among the total of 131 cotton rats receiving material, 35 developed pneumonia and lung lesions described as patchy and reddish-gray with maximum intensity of illness at 6–8 days after inoculation. The etiological agent was presumably a filterable virus as large as 180–250 μm (infectivity was retained by a membrane of an average pore diameter of 300 μm) that differed from the psittacosis-like virus or other known viruses that were known to infect cotton rats by the intranasal route.

Eaton et al. (1944) demonstrated that both infected chick embryo tissues and instillation of infective human material (sputum from 128 persons having atypical pneumonia and lung tissue from 15 patients who had died of the disease) generated similar pulmonary lesions in the animal models of cotton rats and hamsters. In addition, the agent propagated in chick embryos was specifically neutralized by serum from patients who had recovered from primary atypical pneumonia but was not neutralized by acute phase specimens (Eaton et al., 1944).

Eaton (1950) studied antibiotic therapy in his virus-infected cotton rats and described that the agents causing primary atypical pneumonia were sensitive to aureomycin but were somewhat smaller than viruses of the psittacosis-lymphogranuloma group, which were also inhibited by this drug. Unfortunately, however, the virus inoculated into human volunteers was not studied for its ability to grow in chicken embryos, and no inoculations of human volunteers were performed with either the virus propagated in chick embryos or chick embryo lung suspensions infected with the Eaton agent. Thus, the organism was identified in Eaton et al. (1944) but its taxonomy remained obscure until the early 1960s when it was clearly identified as a bacterium.

## Pinehurst Trials

During World War II, management of atypical pneumonia was a serious problem for the United States Army, and the Commission on Acute Respiratory Diseases of the Armed Forces Epidemiological Board performed a series of experiments to investigate this problem (Commission on Respiratory Diseases, 1946d). In October 1943, the Commission on Acute Respiratory Diseases group performed a first transmission study of primary atypical pneumonia to human volunteers (Commission on Acute Respiratory Diseases, 1945) at Fort Bragg, North Carolina, so-called Pinehurst area and demonstrated that unfiltered throat washings and sputa obtained from patients early in the course of the disease caused a respiratory illness in 10 of 12 volunteers.

Next, second and third transmission experiments were conducted during the summer of 1944 (Commission on Respiratory Diseases, 1946a,b,c). The inoculum consisted of throat washings and sputum from patients admitted to Fort Bragg Regional Hospital with atypical pneumonia. Inoculation material was arranged into three patterns (untreated, filtered through Corning sintered glass or Seitz filters, or autoclaved at 15 pounds pressure for 10 min), which was introduced into the nose and throat of each volunteer in synchronization with deep inspiration by means of an atomizer and nebulized three times in a single day.

In the second experiment, each group comprised 12 men, and primary atypical pneumonia occurred equally in the filtered (*n* = 4, 33.3%), untreated (*n* = 3, 25%), and autoclaved (*n* = 3, 25%) groups. The latter group was considered to be due to either contamination of the inner surface of the air pump or cross infection after inoculation.

The third experiment consisted of an autoclaved group (*n* = 18), filtered group (*n* = 12), and untreated group (*n* = 12). The resulting incidence of primary atypical pneumonia in each group was 0%, 25% (*n* = 3), and 25% (*n* = 3), respectively. No cases of pneumonia developed in healthy volunteers who received autoclaved inoculum using rigid precautions during inoculation. The members of the Commission on Acute Respiratory Diseases concluded that the bacteria-free filtrates, presumably containing a virus, could induce primary atypical pneumonia in human volunteers.

Of note, they did not perform the following experiments: (1) direct inoculation of Eaton agent to volunteers, (2) analysis of inoculation materials obtained from patients with primary atypical pneumonia, (3) preinoculation and postinoculation sera from the Pinehurst volunteers in the chick embryo lung/hamster neutralizing antibody assay, (4) determination of cross immunity to Eaton agent in patients with pneumonia in the Pinehurst trial, or (5) neutralization by pretreatment with rabbit antisera to the Eaton agent in further volunteer experiments.

Thus, the failure of collaboration in 1944 between the Commission on Acute Respiratory Diseases members (Dingle, et al.) and the Eaton group left the full significance of the Pinehurst transmission experiments unrealized for 20 years (**Table 1**).

**Table 1:** History of acceptance of the Eaton agent as a cause of primary atypical pneumonia.

Author	Summary
Dienes and Edsall (1937)	First isolation of *Mycoplasma (Mycoplasma hominis)* from humans
Reimann (1938)	Recognition of symptoms of “atypical pneumonia”
Eaton et al. (1944)	Discovery of Eaton agent
Commission on Acute Respiratory Diseases (1945) directed by Dingle et al.	Pinehurst trials: first trial
Commission on Respiratory Diseases (1946b,c) directed by Dingle et al.	Pinehurst trials: second trial
Commission on Respiratory Diseases (1946b,c) directed by Dingle et al.	Pinehurst trials: third trial
Liu et al. (1959)	Establishment of IF technique
Liu et al. (1959)	
Chanock et al. (1960a)	Eaton agent causes lower respiratory tract infection
Chanock et al. (1960b)	Eaton agent grow in cell culture, monkey kidney tissue culture
Chanock et al. (1961b)	Eaton agent causes lower respiratory tract infection
Clyde et al. (1961)	Fluorescent-stainable antibody to the Eaton agent positive for primary atypical pneumonia
Marmion and Goodburn (1961)	Eaton agent is not a virus
NIH conference (1961)	Acceptance of Eaton agent as a cause of primary atypical pneumonia
Rifkind et al. (1962)	Inoculation of volunteers with Eaton agent
Chanock et al. (1962a)	Successful culture of the Eaton agent on cell-free medium
Chanock (1963)	Taxonomic designation of *M. pneumoniae*


## Serological Approaches for Identification of the Eaton Agent

### Cold Hemagglutinins

Peterson et al. (1943) reported that the development of cold agglutinins could serve as a criterion for segregating some of the prevalent cases of primary atypical pneumonia until definite etiological agents could be established. The maximum titer of cold agglutinins (in most cases 1:160 or 1:320 at 0°C) was usually obtained at or near the end of the febrile period, and a rapid decline in titer occurred during convalescence. Dingle and Jordan demonstrated a rise in the titer of cold hemagglutinins in over 80% of inoculated healthy volunteers who had atypical pneumonia or a minor respiratory illness, but the titer was elevated in only one of the patients who did not develop an illness (Commission on Respiratory Diseases, 1946c).

Moreover, correlation of maximum cold hemagglutinin titers with (1) extent of pulmonary involvement, (2) height and duration of fever (Meiklejohn, 1943), and (3) other indices of severity of illness showed no constant trends. Furthermore, Cook et al. (1960) reported that both the hemagglutinin test and streptococcus MG agglutinins frequently failed to develop in patients with atypical pneumonia if the fluorescent antibody test for the Eaton agent was positive.

### Streptococcus MG Agglutinins

Serum streptococcus MG agglutinins will rise in some cases of primary atypical pneumonia. However, the Pinehurst trial (Commission on Respiratory Diseases, 1946c) showed that a rise in the titer of agglutinins for streptococcus MG was not associated with primary atypical pneumonia.

### Neutralizing Antibody for Eaton’s Pleuropneumonia-Like Organisms

Convalescent-phase sera from patients with infections caused by Eaton’s pleuropneumonia-like organism (PPLO) had the ability to inhibit growth of the organism (Eaton et al., 1945; Clyde, 1963). However, this test has little diagnostic role in most instances (Horsfall et al., 1943).

### Fluorescent-Stainable Antibody for Eaton’s PPLO

Liu (1957) described a technique which provided greater facility in making a serologic diagnosis of Eaton agent-related infections. Unlike cold hemagglutinins, fluorescent-stainable antibody elevations develop in the 3rd–4th week of illness, persist for 12–18 months, and appear to be quite sensitive and specific (Liu et al., 1959). In 1960, among patients with primary atypical pneumonia, Cook et al. (1960) established a rise in Eaton fluorescent antibody (FA) titer in 85% of 26 patients with cold and/or streptococcus MG agglutinins and in 26% of 69 patients without cold agglutinins.

### Complement Fixation with Eaton’s PPLO

Lind et al. (1997) studied the diagnostic yield of the Complement Fixation (CF) test using serum samples from an over 50-year period from 1946 to 1995 in Denmark and reported that the sensitivity and specificity of the CF test were 78 and 92%, respectively, if the patient was considered to have a current or recent *M. pneumoniae* infection when the *M. pneumoniae* CF test demonstrated a ≥fourfold rise in titer to ≥64 in at least two consecutive sera (Lind et al., 1997).

## Visualization of the Eaton Agent (1960s)

For many years, the agent was considered to be a virus. However, Marmion and Goodburn (1961) successfully visualized the small coccobacillary bodies on the mucous layer covering the bronchial epithelium of the Eaton agent-infected chick embryo, which suggested that the Eaton agent was not a virus.

Chanock et al. (1960b) demonstrated that propagation in a tissue culture system was possible, but they were unable to visualize the agent directly. In this regard, Clyde et al. (1961) was able to subculture the tissue culture materials obtained from infected chick embryos into monkey kidney cells and finally visualized the brightly stained, rounded, granular structures using Liu’s indirect fluorescent antibody procedure (Liu, 1957). They appeared quite similar to those of the PPLO family.

**FIGURE 2:**
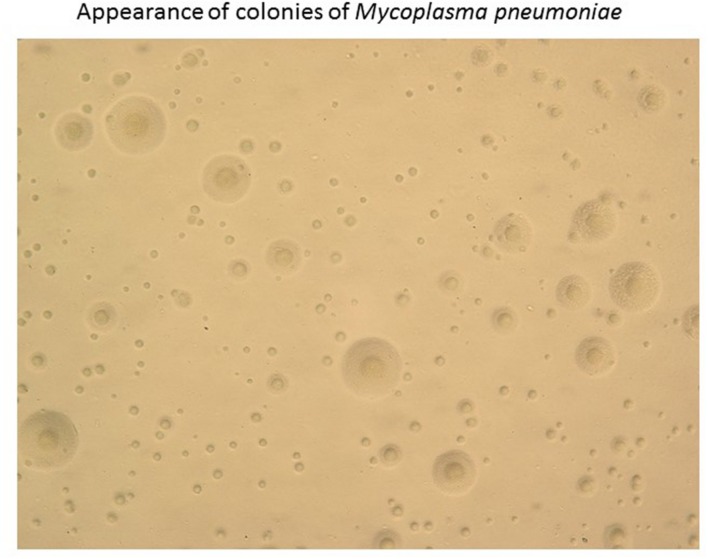
**Appearance of colonies of *Mycoplasma pneumoniae.*** Colonies of *M. pneumoniae* on an agar plate typically have a unique “fried egg” appearance.

Next, Chanock et al. (1962a) described the successful growth of the Eaton agent in cell-free media, incorporating 2.5% yeast extract and 20% horse serum. The colonies that formed on agar were granular, with the center embedded, which occasionally presented as a “fried egg” appearance (**Figure 2**) with a dense center and a less dense periphery.

Taken together, the properties previously defined for the Eaton agent included the following points: (1) size between 180 and 250 μm, (2) sensitivity to tetracyclines and organic gold salts, and (3) occurrence of coccobacillary bodies on infected chick embryo bronchial epithelium. These characteristics were consistent with the contention that the organism was of the PPLO genus. This accumulated evidence strongly suggested that the Eaton agent was a member of, or shared many properties with, the genus *Mycoplasma*.

## Re-Evaluation of the Eaton Agent as a Possible Cause of Primary Atypical Pneumonia via Transmission Experiments to Human Volunteers

After the Pinehurst trials, the stalemate over the acceptance of the evidence was eventually overcome by observations of the nature of the Eaton agent or virus and its recognition as a mycoplasma (Marmion, 1990). However, Chanock et al. (1961b) recovered the Eaton agent with monkey and human kidney tissue and the transmission study was performed by coarse spray and instillation into the noses and mouths of 52 healthy adults (21–36 years of age) from the federal prison system. Among 52 volunteers, the Eaton agent infected all 27 seronegative volunteers (fluorescent antibody titer prior to challenge was less than 1:10), and 17 of 25 individuals who possessed antibody (1:10 or greater) prior to the challenge. This suggested that in the second tissue culture passage, the Eaton agent itself was responsible for initiating the sequence of events which led to pneumonia, otitis, or febrile respiratory disease, irrespective of the presence of a positive fluorescent antibody titer for the Eaton agent (Chanock et al., 1961b; Rifkind et al., 1962).

Clyde et al. (1961) examined the preserved sera from 70 volunteers participating in two primary atypical pneumonia transmission experiments (Pinehurst trials) with regard to fluorescent-stainable antibodies to the Eaton agent. He found that fluorescent-stainable antibody responses were associated with cases of primary atypical pneumonia (Clyde et al., 1961).

In other studies, Chanock et al. (1960a) also revealed evidence that the Eaton agent had developed in 16% of patients with etiologically undiagnosed lower respiratory tract illness using fluorescent-stainable antibody (Eaton antibody). Similarly, other reports of Eaton agent–pneumonia in the 1960s showed that the Eaton agent was certainly considered to be a cause of primary atypical pneumonia (Chanock et al., 1961a; Evans and Brobst, 1961; Kingston et al., 1961; Goodburn et al., 1963).

## Taxonomic Designation *M. pneumoniae*

An accumulation of studies have demonstrated evidence that the organism previously known as “primary atypical pneumonia virus” or “Eaton agent” is not a virus (Clyde and Denny, 1963), but rather, a member of the genus *Mycoplasma* (PPLO; Marmion and Goodburn, 1961; Chanock et al., 1962a,b).

## Acceptance of the Eaton Agent as a Cause of Atypical Pneumonia

In 1926, a Conference on Newer Respiratory Disease Viruses, mycoplasmas, and PPLOs was held at the National Institutes of Health (NIH), and Dr. Dingle finally accepted the Eaton agent as the cause of primary atypical pneumonia (USPHS, 1962).

After being convinced of the data as described in the paragraph of “Re-evaluation of the Eaton agent as a possible cause of primary atypical pneumonia via transmission experiments to human volunteers,” Chanock (1963) finally proposed the nomenclature for the atypical pneumonia organism (Eaton agent) as *M. pneumoniae*.

In the history of *M. pneumoniae* pneumonia, acceptance of the Eaton agent as a cause of the disease required nearly 20 years (Clyde, 1993). Most of the pioneers were lonely and belonged to the small scale laboratories, except for the Pinehurst trials which were supported by numerous workers already well known. Therefore, the delay of acceptance of the Eaton agent possibly due to institutional or group competitiveness (Marmion, 1990), and turned out to be a long journey for Dr. Eaton.

## Author Contributions

TS generated the manuscript and figures.

## Conflict of Interest Statement

The author declares that the research was conducted in the absence of any commercial or financial relationships that could be construed as a potential conflict of interest.
